# Donor-specific immunomodulation is mediated by mechanisms beyond CD4^+^ regulatory T cells

**DOI:** 10.1177/09636897261473440

**Published:** 2026-08-03

**Authors:** Makiko Kumagai-Braesch, Nils Ågren, Masaaki Watanabe, Bo-Göran Ericzon, Michael Uhlin, Ming Yao

**Affiliations:** 1 Department of Clinical Science Intervention and Technology, Division of Transplantation Surgery, 27106 Karolinska Institutet, Huddinge, Sweden; 2 ME Transplantation, Karolinska University Hospital, Huddinge, Sweden; 3 Department of Surgery, Hokkaido University, Sapporo, Japan; 4 Center for Hematology and Regenerative Medicine (HERM), Department of Medicine, 27106 Karolinska Institutet, Huddinge, Sweden

**Keywords:** Treg, donor specific immunomodulatory cells, transplantation, cell therapy, tolerance

## Abstract

Donor-specific immunomodulatory cells (DSIMC) have successfully induced donor-specific tolerance in clinical living donor liver transplantation. These cells are generated from recipient peripheral blood mononuclear cells (PBMCs) stimulated with irradiated donor PBMC in the presence of costimulatory blockade, yielding a heterogeneous population of recipient-derived immune cells including T cells, B cells, and NK cells, in which CD4^+^ regulatory T cells (Tregs) are thought to play a central role. Here, we examined the cell populations and functions crucial for donor-specific immunomodulation within the DSIMC product. DSIMC were generated from PBMC of healthy volunteers using belatacept to achieve costimulatory blockade. Tregs were enriched by CD4^+^CD25^+^ magnetic-cell activated cell sorting (MACS) or CD25^+^CD127^-/low^ fluorescence-activated cell sorting (FACS). CD4^+^, CD8^+^, CD19^+^, and CD25^+^CD127^-/low^ fractions were also individually depleted from DSIMC. Immunomodulatory function of each resulting population was assessed by tritium-labelled thymidine incorporation in mixed lymphocyte cultures (MLC), and cytokine-producing capacity was evaluated by ELISA/ELISpot assays. Unsorted DSIMC exhibited donor-specific suppression at lower cell numbers than CD4^+^CD25^+^ enriched cells, while CD4^+^CD25^-^ T cells showed no immunosuppressive effect. Depletion of CD4^+^, CD8^+^, or CD19^+^ cells did not substantially impair immunomodulatory function. In contrast, depletion of the CD25^+^CD127^-/low^ fraction reduced inhibitory function and abolished donor specificity. Depletion of CD4^+^ or CD25^+^CD127^-/low^ reduced IFN-γ and IL-10 production in response to donor stimulation compared with unsorted DSIMC, implicating CD4^+^ Tregs as important contributors to cytokine production under allogeneic stimulation. Furthermore, Treg expansion following donor antigen restimulation was observed in unsorted DSIMC but not in CD4^+^CD25^+^-enriched cells, suggesting that Tregs can be induced from non-Treg CD4^+^ populations within the DSIMC. In conclusion, CD4^+^CD25^+^CD127^-/low^FoxP3^+^-associated cells appear to contribute to donor-specific immunomodulation; however, the enrichment of CD4^+^CD25^+^ T cell diminished DSIMC efficacy. Since the unsorted DSIMC showed stronger activity, we suggest that multiple cell populations may act together to mediate the DSIMC effect.

## Graphical abstract

**Graphical Abstract:**
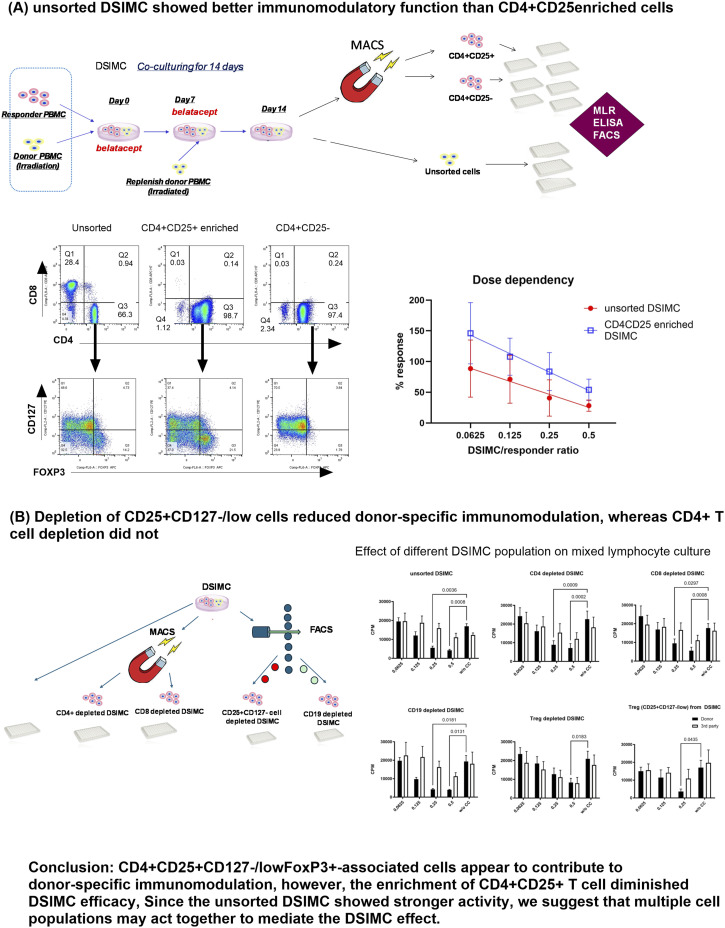

## Introduction

The lifelong immunosuppressive therapy following allogeneic solid organ transplantation carries considerable risk of serious side effects, including chronic kidney disease, opportunistic infections, cardiovascular disease, and malignancy.^1–3^ The prospect of tolerance induction, enabling reduction or cessation of immunosuppression while maintaining graft function, has therefore been a central ambition since the earliest era of solid organ transplantation. Over the past two decades, experimental approaches to tolerance induction have yielded promising results, shifting the field from theoretical possibility toward potential clinical practice.^4,5^ Partial myeloablative conditioning followed by hematopoietic stem cell transplantation (HSCT) to establish transient donor-specific chimerism alongside solid organ transplantation has shown encouraging outcomes.^6^ However, this approach has yet to demonstrate convincing effects in liver transplantation,^7^ and the risks associated with conditioning regimens and graft-versus-host disease may render it unsuitable for frail patients with end-stage liver disease.

More recently, promising results from tolerance induction and immunosuppression withdrawal have emerged in liver transplantation through regulatory cell therapy. This approach aims to utilize the recipient’s own peripheral blood mononuclear cells, conditioning them *ex vivo* to promote immunological acceptance of the allogeneic organ prior to reinfusion.^8,9^ Several transplantation centers are currently developing protocols for tolerance induction after solid organ transplantation using this strategy.^10,11^ However, the immunological mechanisms underlying tolerance induction remain considerably less well understood than those driving rejection.

Since the 1970s, immunomodulatory T cells have been thoroughly studied^12^ and long considered the primary mediator in developing immunological tolerance.^4,13–15^ In the mid-1990s, Sakaguchi et al. demonstrated that a subpopulation of CD4^+^ T cells expressing high levels of the IL-2 receptor α-chain (CD25) plays a critical role in mediating tolerance.^16^ The subsequent identification of the nuclear factor FoxP3 as a functional marker of immune regulation further consolidated the central role of CD4^+^CD25^+^FoxP3^+^ regulatory T cells (Tregs) in this process.^17^ Over the past decade, however, it has become increasingly recognized that other immune cell populations may also contribute to tolerance.^11,18–20^

Regulatory dendritic cells (DCreg), characterized by high expression of inhibitory molecules (PD-L1/2), low expression of costimulatory molecules (CD80, CD86), and secretion of immunosuppressive cytokines (IL-10 and TGF-β), have been shown to modulate antigen-specific T cell tolerance.^21^ FoxP3^+^CD8^+^ regulatory T cells have been implicated in cancer, autoimmunity and transplantation, though the mechanisms governing their development and function remain incompletely understood.^22^ Regulatory B cells have been reported to suppress immune responses and maintain autotolerance through production of anti-inflammatory cytokines including IL-10, TGF-β and IL-35.^23^ CD56 bright NK cells have also been shown to exhibit regulatory functions in maintaining tissue homeostasis.^24^ Beyond these established populations, Type 1 regulatory T cells (Tr1), regulatory subsets characterized by IL-10 and IFN-γ co-production in the absence of constitutive FoxP3 expression, have also been proposed as contributors to peripheral tolerance and allograft acceptance.^25^

Todo et al. reported successful immunosuppression (IS) withdrawal in 7 out of 10 recipients of living donor liver transplantation.^8,9^ Although the cell product was initially described as a regulatory T cell product, it is in fact consisted of unsorted recipient PBMCs stimulated with donor antigens in the presence of costimulatory blockade, administered without any enrichment or purification process.^9^ To date, this product remains one of very few clinically successful immunomodulatory cell therapies that have enabled complete cessation of IS. Clarifying which cell type or types are responsible for tolerance induction in such products is therefore of considerable importance for the further development of this therapeutic approach. In the course of developing a tolerance induction protocol for recipients of deceased donor liver transplantation, we previously described a method to generate donor-specific immunomodulatory cells (DSIMC) using GMP-approved reagents.^26^

In the current regulatory landscape, cell therapies are classified as advanced therapy medicinal products (ATMPs), governed by medicinal product agencies with a general preference for well-defined, single cell line or compound-based products that produce a defined effect.^27–29^ From a regulatory and manufacturing perspective, enrichment or purification of a target cell population is therefore a preferred approach, and many protocols focus accordingly on expanding or purifying a single desired cell subset.^30,31^ However, the immune system is a complex, interdependent network of cells and signals. Immune responses, whether directed toward rejection or tolerance, emerge from coordinated interactions between antigen-presenting cells, T cells, and downstream effector populations, modulated by costimulatory signals and the surrounding cytokine milieu, ultimately giving rise to clonal expansion and crosstalk with humoral immune components.^32,33^ Tolerance induction may, like rejection, depend on the coordinated interaction of multiple cell populations, an equilibrium of regulatory cell types working in concert to establish immune homeostasis.^34^ The mechanistic pathway to tolerance induction remains incompletely understood, and it remains an open question whether purification of a single regulatory subset can recapitulate the efficacy of more complex mixed cell products.

In this study, we aimed to define which cell populations within the generated DSIMC product are responsible for its donor-specific immunomodulatory function. To this end, we compared the immunomodulatory capacity of the intact DSIMC product with that of subpopulations enriched for or depleted of specific cell types, including CD4^+^ T cells, CD8^+^ T cells, B cell, and the CD25^+^CD127^-/low^ Treg population.

## Materials and methods

### Human cell and serum collection

Human peripheral blood was collected from healthy volunteers. The regional Ethics Committee in Stockholm, Sweden, approved the study, including the consent process (Dnr 2014/1565-31/4).

### PBMC separation

Peripheral blood was collected in tubes containing Acid Citrate Dextrose (ACD) solution. Mononuclear cells were sorted by gradient centrifugation using Lymphoprep (Stemcell™ Technologies, Cambridge, UK), as described previously.^26^ Blood group-compatible and HLA-mismatched individual cells were used as stimulators and irradiated with 35 Gy (x-ray irradiator). Specific donor stimulator cells or third-party stimulator cells were frozen with 10% dimethyl sulfoxide (DMSO; Cryosure-DMSO, WAK-Chemie Medical GmbH, Steinbach/Ts.Germany) and thawed immediately before use.

### Human serum

Peripheral blood, collected without an anticoagulant, was stored for 30 minutes at 4°C, after which the aggregate was removed. The serum was heat-inactivated in a 56°C water bath for 30 minutes, and centrifuged at 2500 x g for 20 minutes at 4°C. The supernatant was aliquoted into 1 ml vials and stored frozen at -20°C until use.

### Culture medium

RPMI-1640 supplemented with HEPES (25 mM) and L-Glutamine (2 mM) (Gibco, Thermo-Fisher Scientific) was used for culturing human PBMCs. Belatacept (supplied by Bristol-Myers Squibb AB, Lawrenceville, NJ, USA) and 1% heat-inactivated autologous serum were added to the culture medium to generate immunomodulatory cells.

### Generation of donor-specific immunomodulatory cells (DSIMC)

DSIMC were generated using a procedure described previously.^26^ Responder PBMC (50x10^6^ cells/flask) were cocultured with irradiated (35 Gy) stimulator PBMC (20 x 10^6^cells) and belatacept (40 µg/10^6^ cells) in 15 ml culture medium supplemented with 1% responder serum in 25 cm^2^ culture flasks (Corning, NY). Cells were incubated at 37 °C in a humidified atmosphere with 5% CO_2_. On day 7, the culture medium was exchanged, and stimulator PBMC and belatacept were replenished. The generated cells were collected on day 14 and used for further analysis.

### Flow cytometry

**Figure 1.**
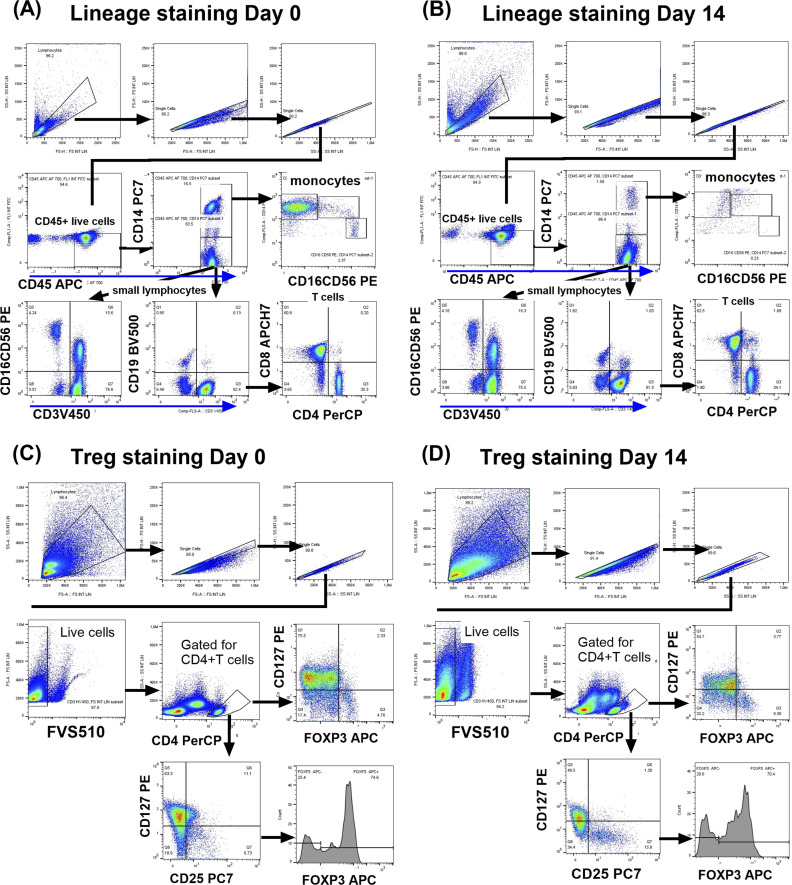
Gating strategies for lineage staining (A and B) and Treg staining (C and D). (A) and (C) showed cells from day 0, and (B) and (D) show cells generated after 14 days of culture. (A, B): Cell were gated for lymphocytes by SS-A/FS-A, then single cells were separated by using FS-H/FS-A, then SS-H/SS-A. CD45+ live cells were gated by FL-1/CD45-APC, then, according to CD14 positivity, cells were separated between small lymphocytes and monocytes. Small lymphocytes were separated CD19^+^ B cells, CD3^+^ T cells, CD16^+^ CD56^+^/CD3^-^ NK cells and CD16^+^ CD56^+^/CD3^+^ NKT cells. CD3^+^ T cell population was further separated CD4^+^, CD8^+^, CD4CD8 both positive, and both negative. population. (C and D): Live lymphocytes were gated by FS-A and SS-A, then gated for single cells. Live cells were selected by FS-A and FSV510 negative. CD4^+^ T cells were separated and the CD127^-/low^ FOXP3^+^, or CD127^-/low^CD25^+^ population and then examined for FOXP3^+^ population.

Freshly isolated responders or generated cells were stained and analyzed using flow cytometry on days 0, and 14. For examination of lymphocyte and monocyte lineage markers, the following conjugated antibodies (Abs) were used: anti-CD4-PerCP Cy5.5, anti-CD8-APC H7, anti-CD3-HV450, and anti-CD19-HV500; and anti-CD16-PE, anti-CD56-PE, (from Beckman Coulter), anti-CD14-PC7, and anti-CD45-APC AF700 (BD Bioscience). Tregs were identified using anti-CD127-PE(BD Biosciences), anti-CD4-PerCP Cy5.5, anti-CD25 PC7 (Biolegend, San Diego, USA), and anti-FoxP3-AF647, clone 236A/E7 (BD Biosciences). The staining procedure, including fixation and permeabilization, followed the manufacturer’s instructions (eBiosciences, San Diego, CA, USA). To prevent non-specific binding, mouse immunoglobulin G1 was added before incubation with the FoxP3 antibody. Data were acquired using a Navios flow cytometry instrument and analyzed with Kaluza software (Beckman Coulter), or FACSdiva (version 8.0.2, BD bioscience) for cell separation. In a previous study, we showed that the frequency of CD4^+^CD25^+^CD127^-/low^FoxP3^+^ T cells increased from 4.1±1.0% (preculture) to 7.3±2.6% (day 14) in belatacept treated groups (p<0.05).^26^ Beyond this, the overall cell composition before culture and at day 14 was examined and found to be broadly similar between timepoints. The gating strategy of freshly isolated PBMC and 14 days cultured cells is shown in Figure 1.

### Magnetic-activated cell sorting (MACS)

Tregs were enriched using the CD4^+^CD25^+^ Regulatory T Cell Isolation Kit from Miltenyi Biotech AB (Lund, Sweden). Briefly, CD4^+^ T cells were isolated by negative selection, followed by positive selection of CD25^+^ cells using CD25 microbeads, according to the manufacturer’s instructions. The resulting product contained approximately 95% CD4^+^ T cells, with CD25^+^ cells comprising up to 75% of the enriched fraction.

Depletion of CD4^+^ or CD8^+^ T cells was performed using anti-CD4 and anti-CD8 microbeads (Miltenyi Biotech) and dead cells were removed using a Dead Cell Removal Kit (Miltenyi Biotech), both according to the manufacturer’s instructions. The resulting products contained approximately 90% viable cells. Purity of the sorted populations was confirmed by flow cytometry using the gating strategy shown in Figure 1, with residual contaminating cells comprising less than 5% of the final product, as shown in Figure 3(b).

### Fluorescence-activated cell sorting (FACS)

Cultured cells were washed with PBS and stained with Fixable Viability Stain 520 (BD Biosciences, NJ, USA) following the manufacturer’s instructions. Cells were then stained with anti-CD25-PC7 (clone BB515, BioLegend, CA, USA) and anti-CD127-PE (clone hIL_7R-M21, BD Biosciences). In preliminary observations, the majority of the Treg population (CD25^+^CD127^-/low^FoxP3^+^ cells) within the generated cell product was found to reside in the CD4^+^ T cell fraction. To avoid unnecessary antibody binding on collected non-Treg population, CD25^+^CD127^-/low^ cells were therefore depleted without prior CD4^+^ labelling. The cell sorting strategy is shown in Figure 3(c).

For CD19^+^ B cell depletion, generated cells were stained with Fixable Viability Stain 520 (BD Horizon™) to discriminate live and dead cells, followed by labeling with anti-CD19-BV510 (clone HIB19, BD Biosciences). CD19^+^ cells were then excluded from the live cell gate to yield the non-B cell population. Cell sorting was performed at the Core Facility of Karolinska Institutet, Stockholm, Sweden, using a FACSAria with FACSDiva software (BD Biosciences), as shown in Figure 3(c) and (d).

### Stimulation of generated cells *in vitro*

On day 14, generated cells were harvested and subjected to CD4^+^ T cell separation and Treg enrichment using MACS microbeads as described above. The sorted populations were then co-cultured with irradiated donor PBMCs in 48-well plates at 10^6^ cells per well to assess donor antigen-specific restimulation. Following culture, cells were harvested and cell composition was examined by flow cytometry.

### Proliferation assays

To assess the suppressive function of the generated cells, their effect on mixed lymphocyte culture (MLC) responses was evaluated by measuring proliferation of responder PBMCs co-cultured with irradiated allogeneic PBMCs. Freshly isolated, antigen-inexperienced responder PBMCs, obtained from the same donor as the generated cells, were seeded at 10^5^ per well in 96-well U-bottomed microculture plates. Irradiated stimulator cells derived from either the specific donor or third-party individuals were added simultaneously with generated cells at decreasing concentrations of 5×10^4^, 2.5×10^4^, 1.25×10^4^, or 0.625×10^4^ cells per well, in a total volume of 0.2 mL per well of medium supplemented with 1% autologous responder serum.

*1) Tritium-labelled thymidine incorporation tests*

Proliferation was assessed by tritium-labelled thymidine incorporation on day 7. On day 6, 1 µCi of tritium-labelled thymidine (PerkinElmer, Shelton, CT) was added to each well and incorporated into the DNA of dividing cells over an 18-hour incubation period, after which cells were harvested. Tritium- labelled thymidine incorporation was indicated by a MicroBeta counter (PerkinElmer). To distinguish allogeneic proliferative responses from background incorporation due to spontaneous proliferation or apoptotic cells, control wells were established consisting of responder PBMCs co-cultured with irradiated autologous stimulator cells, responder PBMCs alone, and irradiated stimulator cells alone, which exhibited <800 cpm, <300 cpm and <100 cpm, respectively. Allogeneic MLC without generated cells exhibited approximately 20,000 cpm, confirming that high thymidine incorporation reflected allogeneic responses rather than background activity.

*2) Ki67 staining*

To examine which cell populations were affected by co-culture with DSIMC, cells were harvested on day 7 from 96-well culture plates as described above, using responder-to-DSIMC ratios of 1:0.5, 1:0.25, alongside responder cells cultured alone as a control. Harvested cells were transferred to FACS tubes and stained with Fixable Viability Stain 510 (BD Horizon™) to discriminate live and dead cells, followed by surface staining with anti-CD4-PerCP-Cy5.5, and anti-CD8-APC-H7. Cells were then fixed and permeabilized, treated with Fc blocker, and stained intracellularly with FITC-conjugated anti-Ki67 antibody (BD bioscience). Stained cells were acquired on a FACSCanto instrument and analyzed using FlowJo software version 10.10.0 (BD Bioscience).

### Cytokine profiles

Experimental co-cultures were established as described above (see Proliferation assays). Culture supernatants were harvested on day 6 and analyzed for IFN-γ and IL-10 concentrations by enzyme-linked immunosorbent assay (ELISA) using kits obtained from Mabtech AB (Nacka, Sweden). Recombinant IFN-γ and IL-10 (Peprotec Inc., NJ, USA) were used to generate standard curves for quantification.

DSIMCs depleted of different populations were also restimulated by co-culturing with irradiated donor antigens and then cytokine production was examined by ELISpot according to the manufacturer's protocol (Mabtech AB.) Spot-forming units were detected by IRIS (Mabtech AB).

### Statistical analysis

For pairwise comparisons, Wilcoxon matched-pairs signed rank tests were used. For three or more groups, one-way ANOVA with Tukey’s multiple comparisons test or two-way ANOVA with Dunnett’s multiple comparisons tests was applied as appropriate. A p-value of <0.05 was considered statistically significant. All statistical analyses were performed using GraphPad Prism® software version 10.6 (GraphPad Software Inc., San Diego, CA, USA).

## Results

### Non-sorted DSIMC showed more effective donor-specific immunomodulation than Treg-enriched cells

**Figure 2.**
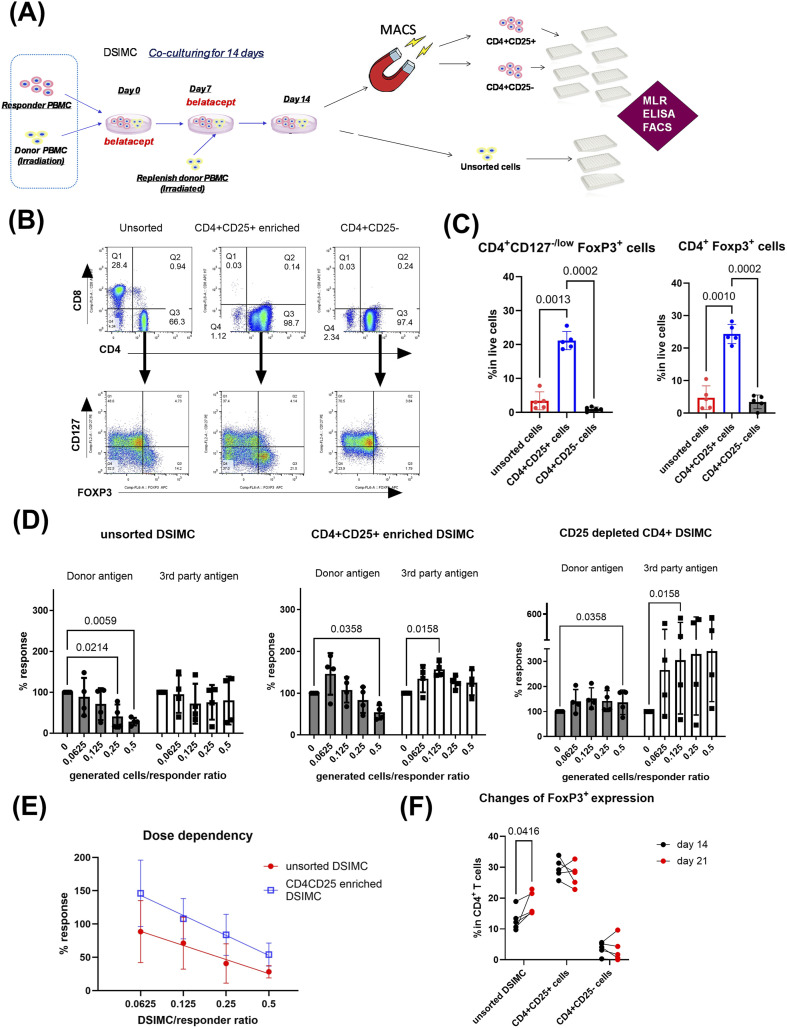
Effect of Treg enrichment by MACS beads. (A) Experimental schema of generated cells using PBMC from healthy volunteers. Responder PBMC were co cultured with irradiated donor PBMCs and belatacept for total 14 days. The collected cells were separated into (1) unsorted group, (2) the CD4^+^CD25^+^, and (3) CD4^+^CD25^-^cell populations. (B) One example of CD4^+^ T cell population in DSIMC and gating methods for CD127^-/low^FOXP3^+^ populations of CD4 gated cells. (C) The percentage CD127^-/low^FOXP3^+^ CD4^+^ cells (left figure), and FoxP3^+^ CD4^+^ cells (right figure) in live cells of each group was compared. The statistical analysis was performed with one-way ANOVA and Tukey’s multiple comparisons test. n=5. (D) Unsorted, CD4^+^CD25^+^ enriched, and CD4^+^CD25^-^ DSIMC were added to MLC with either specific donor stimulators or 3rd party stimulators. Relative proliferation was compared in each group against cell proliferation without DSIMCs. Left: The co-culture with the non-separated DSIMC showed a reduction of proliferation in MLC with the specific donor stimulator in a dose-dependent manner, while no effect was observed in MLC with the 3rd party stimulator. Middle: CD4^+^CD25^+^ enriched DSIMC was added to MLCs. The addition of low doses of CD4^+^CD25^+^ generated cells slightly enhanced proliferation; however, with higher doses, the proliferation was suppressed in MLCs with specific donor stimulators. Right: the addition of CD4^+^CD25^-^ DSIMC to MLC increased proliferation in MLC with both donor and 3rd party stimulators, most obviously in MLCs with 3rd party stimulators. (E) Donors-specific responses were reduced in a dose dependent manner, and the curve was parallel in both whole generated cells and CD4^+^CD25^+^ T cells. n=5, 2way-ANOVA with Dunnett’s multiple comparison. (F) Expansion of Treg population by donor Ag stimulation. The generated cells were harvested on day 14 and separated into three groups: (1), unsorted generated cells; (2) CD25^+^CD4^+^ enriched T cells. (3) CD4^+^CD25^-^ T cells. These generated cells were cultured with irradiated specific donor cells for one week. The percentage or FoxP3^+^ cells in CD4^+^ T cell population were compared (between day 14 and day 21). n=5. Differences were examined by 2way ANOVA and Šídák’s multiple comparisons test.

To evaluate the immunomodulatory effects of Tregs in DSIMC, generated cells were sorted into CD4^+^CD25^+^ and CD4^+^CD25^-^ fractions using MACS microbeads (Figure 2(a)). Tregs were identified by flow cytometry analysis, defined as CD4^+^CD127^-/low^Foxp3^+^.

The representative examples of CD127 and FoxP3 expression with and without cell enrichment are shown in Figure 2(b). In unsorted cells, CD4^+^ cells comprised 50-60% of the total population, with Tregs accounting for 3-5%. The CD4^+^CD25^+^ enriched fraction consisted of approximately 70% CD25^+^ cells, of which CD127^-/low^ FoxP3^+^ cells accounted for 30-40%, yielding an overall Treg population of approximately 20%. The CD4^+^CD25^-^ fraction contained fewer than 1% CD127^-/low^FoxP3^+^ cells (Figure 2(c) left panel). As CD25 and CD127 are affected by activation and maturation status during culture, and FoxP3^+^ population was found in CD127^+^ cells, % cell population of CD4^+^FoxP3^+^ was also compared (Figure 2(c) right panel). When the broader CD4^+^FoxP3^+^ population was examined, CD4^+^CD25^-^ cells contained 0.2-5% FoxP3^+^ cells, of which <1% were CD127^-/low^ FoxP3^+^. The implications of Treg marker expression under culture conditions are discussed further below.

Unsorted DSIMC, CD4^+^CD25^+^-enriched generated cells, and CD4^+^CD25^-^ cells were added to MLCs at various ratios relative to responder cells, and proliferation was measured on day 7. As shown in Figure 2(d), unsorted DSIMC exerted a clear inhibitory effect on MLC responses to specific donor stimulators at generated cell-to-responder ratios of 0.25 and 0.5. The CD4^+^CD25^+^-enriched fraction also demonstrated suppression at the highest dose, whereas CD4^+^CD25^-^ cells showed no suppressive effect at any ratio. No suppressive effect against third-party stimulators was observed in any group. Donor-specific responses were reduced in a dose-dependent manner, with parallel dose-response curves observed for unsorted DSIMC and CD4^+^CD25^+^ enriched cells (Figure 2(e)). These results suggested that the CD4^+^CD25^+^ enriched fraction retains donor-specific immunomodulatory function, however, enrichment of this population did not improve immunomodulatory efficacy compared to unsorted DSIMC, suggesting that other cell populations within the DSIMC also contribute to this function.

The number of IFN-γ or IL-10-producing cells in unsorted and Treg-enriched DSIMC was examined 24 hours after co-culturing with irradiated donor cells. The Treg-enriched group contained fewer IFN-γ-producing cells and slightly more IL-10-producing cells compared to unsorted DSIMC under donor-specific stimulation (Supplemental Figure 1).

### Donor antigen restimulation expands the Treg population in unsorted DSIMC

The generated cells were harvested on day 14 and divided into two groups: unsorted DSIMC, and CD25^+^CD4^+^ enriched cells. Both populations were cultured with irradiated specific donor cells for seven days, and the proportion of CD127^-/low^ FoxP3^+^ cells or FosP3^+^ within CD4^+^ T cell population was compared between day 14 and day 21.

In non-sorted DSIMC, the CD127^-/low^FoxP3^+^ fraction and FoxP3^+^ fraction within CD4^+^ T cells expanded significantly following donor antigen restimulation (day 14 vs. day 21, p=0.0382 and p=0.0313, respectively, paired t-tests). In contrast, these populations in CD25^+^CD4^+^-enriched cells did not expand further. Within the CD4^+^CD25^-^ fraction, the CD127^-/low^FoxP3^+^ population showed a modest but non-significant increase following restimulation (Table 1). Figure 2(f) shows FoxP3 fractions in CD4+T cells before and after donor antigen stimulation in each group (day 14 vs day 21). These findings suggest that the Treg expansion observed in non-sorted DSIMC following restimulation may derive from the non-Treg CD4^+^CD25^-^ population, consistent with previous reports that peripherally induced Tregs may not constitutively express CD25 or FoxP3.^35^ The relationship between Treg markers and functional identity under culture conditions is discussed further below.

**Table 1.** Changes of FoxP3^+^ or CD127^-/low^FoxP3^+^ populations in CD4^+^ T cells after donor antigen stimulation.

​	FoxP3+ in CD4+ T cells			CD127-/lowFoxP3+ in CD4+ T cells		
	Day 14	Day 21*	p-value	Day 14	Day 21*	p-value
unsorted DSIMC	13.0±3.6	18.2±3.7	p=0.03146	8.9±3.4	15.1±4.1	p=0.03313
CD4+CD25+ DSIMC	29.6±3.1	24.3±3.1	ns	24.3±3.1	23.4±5.8	ns
CD4+CD25- DSIMC	3.7±2.1	3.3±3.0	ns	1.0±0.7	2.4±1.8	ns

Day21* DSIMC cultured with donor antigens for 7 days and Treg marker was examined.

N=5, two groups (day 14 vs day 21) were compared by paired student t-test. ns: not significant.

### Depletion of CD25^+^CD127^-/low^ cells reduced donor-specific immunomodulation, whereas CD4^+^ T cell depletion did not

**Figure 3.**
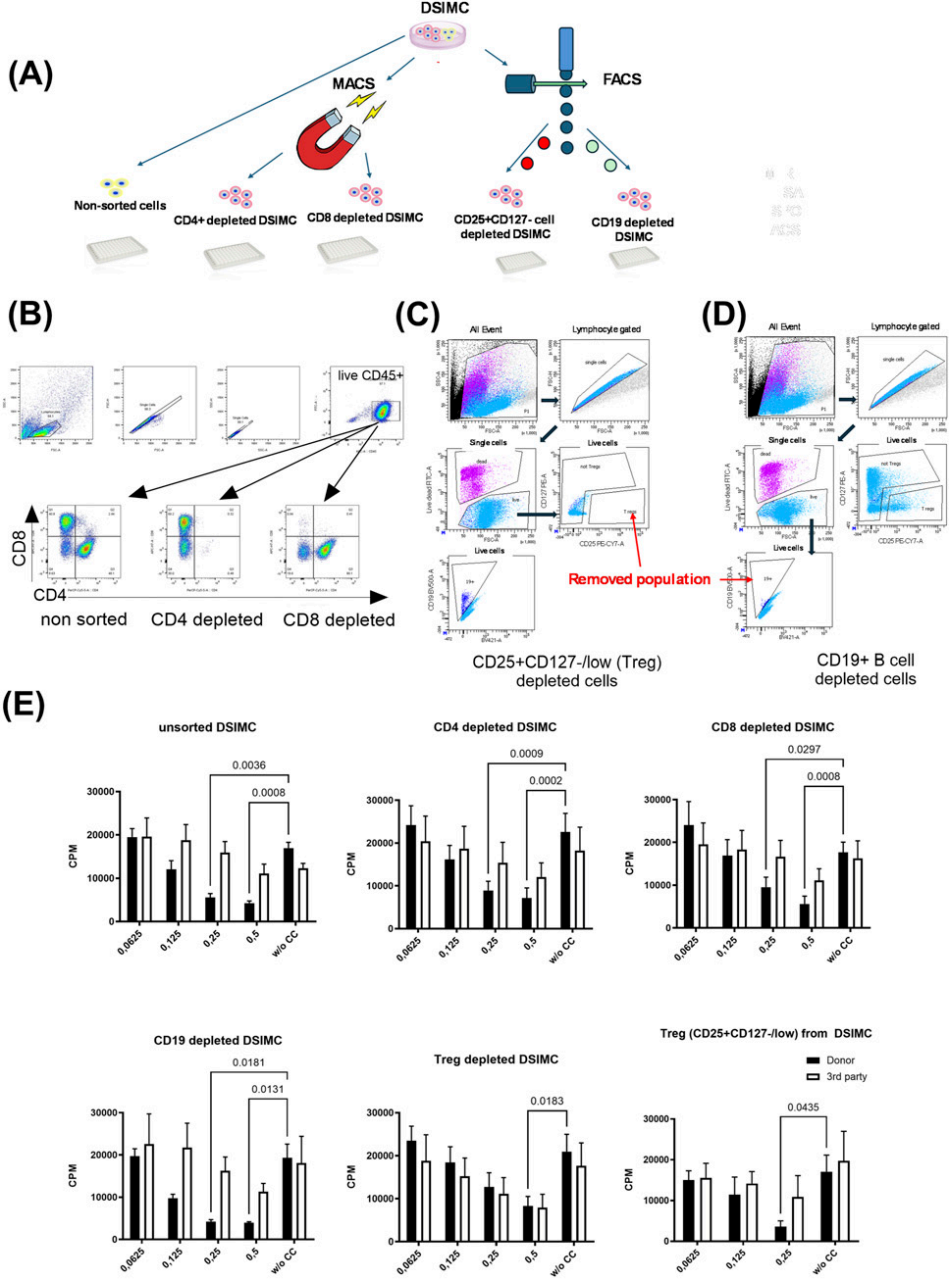
Generated cell sorting methods and function. (A) Shema of cell depletion strategies. DSIMC were grouped as non-sorted, CD4 or CD8 depleted (by MACS), CD19 or CD25^+^CD127^-/low^ (Treg) depleted, and Treg sorted (CD25^+^CD127^-/low^) populations (by FACS). (B) One example of the efficacy of depletion of CD4 or CD8^+^ T cells are shown. Gating strategy by flowcytometry for (C) depleting Treg and (D) CD19^+^ B cells is also shown. (E) Effect of different fractions of generated cells on MLC with donor-specific stimulator and 3rd party stimulators. Mean±SE is shown. n=6, except Treg-depleted-generated cells and Treg-generated cells, which had n=5. Donor-specific proliferation was suppressed in a dose-dependent manner. Suppressive efficacy was similar in non-sorted DSIMC, and CD4-, CD8- or CD19-depleted DSIMC. The Treg depleted population did not significantly reduce proliferation at generated cell/responder ratio of 0.25/1. Treg population showed a significant suppressive effect at the responder/generated cells ratio of 1/0.25 for both specific donor and 3rd party stimulated MLCs. The statistical differences were examined by Two-way ANOVA, Dunnett’s multiple comparison tests.

To further investigate the cell populations mediating the immunomodulatory effect of DSIMC, specific cell populations were depleted as described in the Materials and Methods. Brief methods were illustrated in Figure 3(a) and cell populations are shown flowcytometry pictures. The estimated Treg population (CD4^+^CD25^+^CD127^-/-/low^FoxP3^+^) in each generated fraction is shown in Table 2. Unsorted DSIMC contained 0.8-3.1% Tregs, Treg population in CD4-depleted DSIMC was below the detectable range, and CD8-depleted DSIMC 1.2-4.1%. Within the CD25^+^CD127^-/low^ fraction of cultured DSIMC, FoxP3 was expressed by only 9.4-44.3% (based on the staining of unsorted cells).

**Table 2.** Percentage CD4^+^CD25^+^CD127^-/low^ FoxP3^+^ cells (Treg) in the sorted generated cell fractions.

DSIMC ID	Non-sorted DSIMC	CD4-depleted^*^	CD8-depleted	CD19-depleted	Treg-depleted (CD25+CD127-)	% FoxP3 in Treg-enriched (CD25+CD127-)^#^
^#^1	1.00	<0.1%	1.51	1.01	0.00	25.54
^#^2	1.50	<0.1%	2.19	1.54	0.00	26.93
^#^3	2.81	<0.1%	3.75	3.03	0.00	41.13
^#^4	0.76	<0.1%	1.39	0.78	0.00	31.36
^#^5	3.08	<0.1%	4.08	3.15	0.00	44.33
^#^6	1.27	<0.1%	1.53	1.33	0.00	9.43
Mean	1.74		2.41	1.81	0.00	29.79
SD	0.97		1.20	1.03	0.00	12.52

^*^undetectable, the values are calculated based on non-sorted Treg percentages.

^#^due to the lack of number of cells, analysis was done based on the non-sorted cell analysis.

As shown in Figure 3(e), non-sorted DSIMC suppressed MLC proliferation in a dose-dependent manner, with significant donor-specific immunomodulation at generated cell-to-responder ratios of 0.5 and 0.25. CD4^+^-depleted DSIMC (comprising CD8^+^ T cells, NK cells, and B cells), CD8^+^-depleted DSIMC (comprising CD4^+^ T cells, NK cells, and B cells), and CD19^+^-depleted DSIMC (comprising T cells and NK cells) all reduced donor-specific proliferation at ratios of 0.25 and 0.5, at levels comparable to non-separated DSIMC. CD25^+^CD127^-/low^-depleted DSIMC demonstrated reduced donor-specific suppression, with a significant effect only at the 0.5 generated cell-to-responder ratio. No significant suppression of third-party responses was observed in any group, with the exception of the CD25^+^CD127^-/low^ population, which exerted significant suppressive effects at a 0.25:1 ratio against both specific donor and the 3^rd^-party stimulators, though this effect was not sustained at lower ratios.

The cell populations proliferating during MLC were further examined by Ki67 staining, with co-staining for CD4 or CD8 T cells (Supplemental Figure 2). Notably, generated cells themselves expressed Ki67 at higher levels than culture-naïve responder cells, meaning that the Ki67 data reflect the combined proliferative activity of both responder and generated cell populations. Ki67 expression was broadly similar across non-sorted DSIMC, CD4-depleted DSIMC and CD8-depleted DSIMC at a generated cell-to-responder ratio of 1:0.5, and in Treg-enriched cells at a ratio of 1:0.125. CD4-depleted DSIMC co-cultures showed lower Ki67 expression in the CD4^+^ responder fraction, while CD8-depleted DSIMC co-cultures showed lower Ki67 expression in the CD8^+^ fraction, consistent with the reduction in those populations within the generated cell product. Of note, in two out of three experiments, co-culture with CD19^+^-depleted DSIMC showed was associated with higher Ki67 expression even in the presence of autologous stimulators, and a similar increase was observed following Treg depletion, affecting both CD4^+^ and CD8^+^ T cell fractions. These observations suggest that B cells and Tregs within the DSIMC may contribute to a broader, non-antigen-specific suppressive function, though further investigation is required to confirm this.

**Figure 4.**
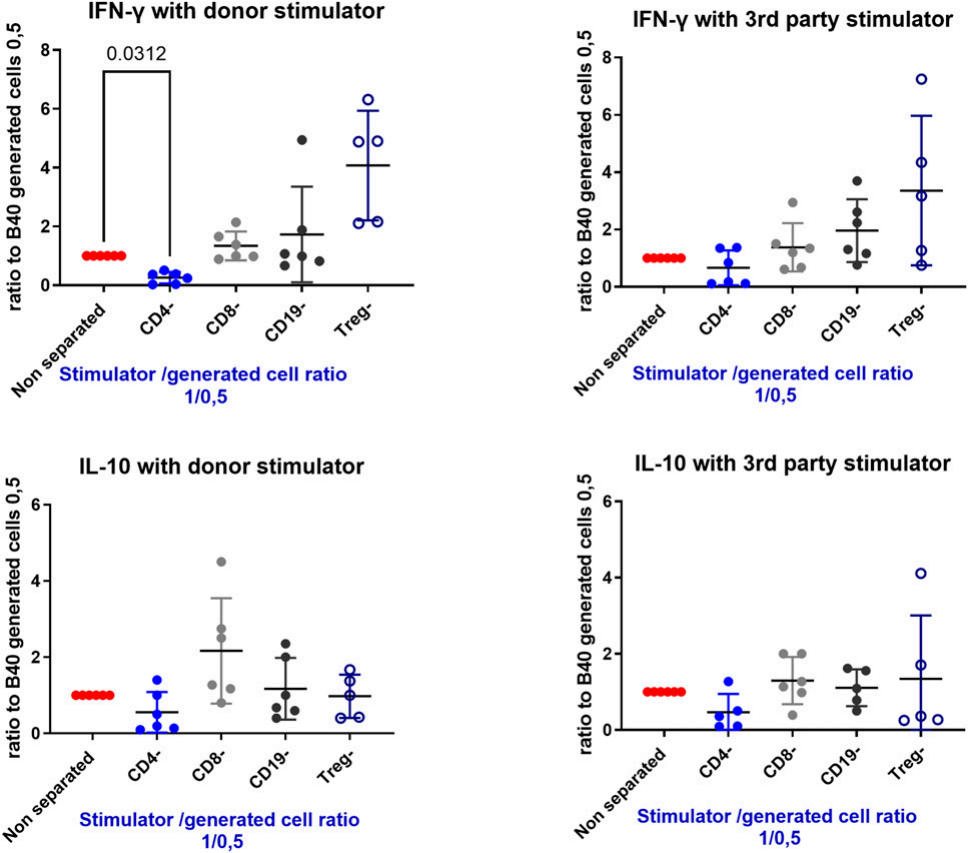
Relative cytokine concentration after 6-day culture of MLC supernatant with different fractions of generated cells. The generated cells were separated into different subgroups and co-cultured with naive responders and irradiated stimulators for 6 days. Culture supernatant was collected, and the cytokine amount was measured by ELISA. Naive responder/generated cell ratio was 1/0.5. Cytokine concentration in culture supernatants with different fractions of generated cells was compared to culture supernatants with unsorted cells. n=6 The two-group comparisons were examined by the Wilcoxon tests.

Cytokine concentrations in culture supernatants were also assessed and compared to MLCs co-cultured with non-sorted DSIMC. CD4-depleted DSIMC significantly suppressed IFN-γ production over 6 days of MLC. Although it did not reach statistical significance, Treg-depleted DSIMC showed a trend toward increased IFN-γ production. CD8-depleted DSIMC, which contained relatively high proportions of CD4^+^ T cells, NK cells, and B cells, produced elevated IL-10 following donor-specific antigen stimulation (Figure 4).

**Figure 5.**
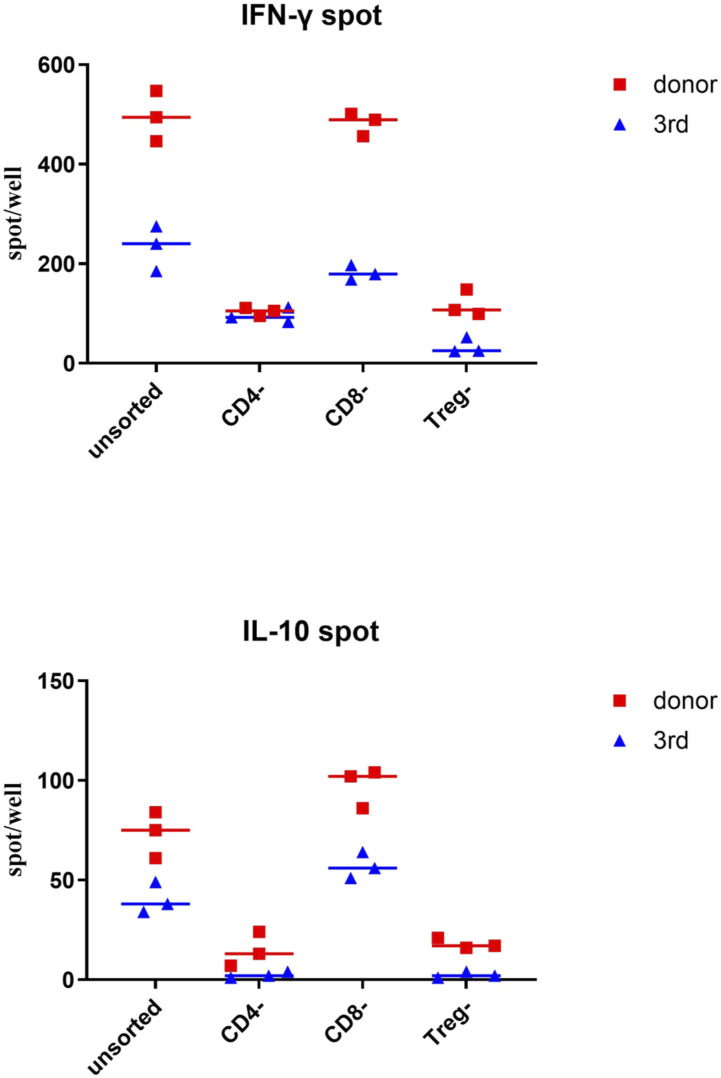
Changes in cytokine-producing cell numbers by fragment depletion from DSIMC. Detection by ELISpot assays. DSIMC were unsorted, depleted of CD4^+^, CD8^+^, or CD25^+^CD127^-^^/low^ (Treg), and stimulated with specific donor cells or 3rd party stimulators. Cytokine-producing cells were spotted after 24 hours of incubation. CD4-depleted and Treg-depleted DSIMC clearly reduced IFN-γ and IL-10 production by donor antigen stimulation. Similar tendencies were observed in the 3rd-party stimulation. (CD4-: CD4-depleted DSIMC. CD8-: CD8-depleted DSIMC. Treg-: CD25^+^CD127^-/low^ depleted DSIMC).

The cytokine-producing capacity of subset-depleted DSIMC was further characterized using ELISpot assays following 24 hours of incubation with irradiated donor or third-party stimulators (Figure 5). Both CD4^+^-depleted and CD25^+^CD127^-/low^-depleted DSIMC showed markedly reduced IFN-γ and IL-10 production following specific donor antigen stimulation, with similar trends observed under 3^rd^-party stimulation. These findings suggest that the CD4^+^ Treg population plays an important role in cytokine production under allogeneic stimulation conditions.

## Discussion

Regulatory cell therapy has shown promise in tolerance induction and minimizing immunosuppression after solid organ transplantation. In this study, we attempted to clarify the importance of different cell types within the DSIMC for donor-specific immunomodulatory effects. CD4^+^CD25^+^-enriched DSIMC showed a clear donor-specific immunomodulatory function, however, their efficacy was inferior to that of non-sorted DSIMC. Meanwhile, CD25-depleted CD4^+^ T cells lost their immunosuppressive ability completely. The FoxP3^+^CD127^-/low^ population in non-sorted DSIMC expanded after restimulation with donor-specific antigens, while they did not in CD4^+^CD25^+^-enriched DSIMC. Depletion of CD25^+^CD127^-/low^ by FACS sorting not only diminished the immunomodulatory effectiveness but also eliminated donor specificity. This suggested that the CD25^+^CD127^-/low^ population (mainly Tregs) may play a crucial role in donor specificity in this cell product. Of note, the CD4^+^ depleted generated cells still maintained their donor-specific immunomodulatory effect, indicating that there might also be an antigen specific immunomodulatory function within the non-CD4^+^ T cell populations. Depletion of CD8^+^ or CD19^+^ cells had a limited impact on DSIMC function in thymidine incorporation assays. Ki67 staining data indicated depletion of cell populations may affect DSIMC function, and in two experiments the addition of CD19 or Treg-depleted population showed higher Ki67 expression on both CD4^+^ and CD8^+^ T cells compared to the group with non-sorted DSIMC. These non-specific proliferative effects were not observed in a donor antigen-specific manner. CD25^+^CD127^-/low^ Treg from DSIMC produced IFN-γ and IL-10 when stimulated with donor antigens. IFN-γ production in MLCs was suppressed by co-culturing with non-CD4 DSIMC but not with the Treg-depleted DSIMC.

Tregs have been viewed as the key component of tolerance induction over recent decades,^36^ and enriched or purified Tregs have often been used in tolerance-induction cell therapy protocols following organ transplantation.^37–39^ Our results confirm that the CD25^+^CD127^-/low^ population within the DSIMC play a crucial role in donor-specific immunomodulation. However, optimal immunomodulatory efficacy was observed with the non-sorted cell product, supporting the theory that multiple interacting cell subsets within the DSIMC collectively underpin donor-specific immunomodulatory function.

After re-challenge with specific donor antigens, non-sorted DSIMC expanded the Treg population (Figure 2(f) and Table 1). This Treg expansion was not clear in the CD4^+^CD25^+^ Treg-enriched population, indicating that the Treg population was induced from the CD4^+^CD25^-^ T cell population in this *in vitro* system. This is in accordance with the previous description that induced T regulatory cells originate from CD4^+^CD25^-^ T cells, and these functions are important to maintain peripheral tolerance.^40–42^ Both CD4^+^CD25^+^ and CD4^+^CD25^-^ populations in the ex-vivo generated DSIMC may be needed to create a tolerogenic environment towards an allograft. Accurate quantification of this Treg expansion is, however, complicated by the phenotypic instability of Treg surface markers in culture conditions. It has been reported that the CD25^+^CD127^-/low^FoxP3^+^ phenotype is more characteristic of thymic or natural Tregs (tTregs/nTregs), whereas peripherally induced Tregs may not consistently express these markers. Cell activation further complicates phenotypic interpretation, as CD25 (IL-2 receptor α chain) is upregulated on activated effector T cells, and CD127 expression is subject to change during peripheral T cell maturation. FoxP3 expression itself may also be transiently downregulated during cell division. Consistent with this, we observed that FoxP3 expression within the CD4^+^CD25^+^CD127^-/low^ population was 75-85% in freshly isolated PBMCs, falling to approximately 40-60% following culture. Given this uncertainty surrounding Treg surface markers in a culture setting, we additionally examined the FoxP3^+^ fraction within the broader CD4^+^ T cell population, rather than relying solely on the CD127^-/low^FoxP3^+^ gate. A proportion of CD127^+^FoxP3^+^ cells was observed; however, the changes in this population before and after stimulation were consistent with those seen using the conventional gating strategy (Figure 2(c) and (f)).

Depleting CD8^+^ cells from the DSIMC did not dampen donor-specific immunomodulatory efficacy in our study. This observation is in line with the fact that the CD8^+^ population mainly consists of cytotoxic effector cells. However, it should be noted that CD8^+^ FoxP3^+^ cell populations with presumed immunoregulatory functions have previously been reported.^22^ As noted above, CD25^+^CD127^-/low^ in both CD4^+^ and CD8^+^ T cell compartments may therefore contribute to donor-specific immunomodulation and tolerance induction.

The cytokine-producing capacity of DSIMC activated by donor antigens was examined with ELISpot (Figure 5). Our results indicate that the CD4^+^ Tregs in the DSIMC could have a major cytokine-producing function following alloantigen stimulation. Both CD4-depleted-DSIMC and CD25^+^CD127^-/low^-depleted cells showed lower IFN-γ and IL-10-producing capacity compared to non-sorted-DSIMC. IL-10 is an anti-inflammatory cytokine, produced by various cells including macrophages, type II helper T cells and Tregs, and is responsible for the creation of a tolerogenic cytokine environment.^43^ IFN-γ is mainly seen as a proinflammatory cytokine produced by various cells, including macrophages, memory T cells, and NK cells upon stimulation by foreign antigens. Tregs reduce the IFN-γ production of effector T cells and macrophages,^44,45^ which in return reduces the efficacy of M1 macrophage differentiation.^46^ Activated Tregs produce high levels of IL-10 and TGF-β, leading to the suppression of IFN-γ production of naive CD4^+^ T cells, which is crucial to maintain peripheral tolerance.^40–42,47^ It is also reported that Tregs produce a low amount of IFN-γ, which it has been suggested to be beneficial for optimal Treg function.^48–50^ IFN-γ has been shown to promote Th1-like Tregs, which in turn suppress cytotoxic CD8^+^ T cell development.^51^ Thus, regulating IFN-γ production may be important for optimal induction of allograft tolerance. While ELISpot analysis provided informative data on the cytokine-secreting capacity of DSIMC subpopulations, it does not permit identification of the precise cellular source of cytokines at the single-cell level. Intracellular cytokine staining (ICS) combined with cell lineage markers would provide more granular mechanistic insight into which populations are responsible for IFN-γ and IL-10 production following donor antigen stimulation. However, reliable ICS requires optimized restimulation conditions including protein transport inhibition with agents such as brefeldin A or monensin, and this analysis was beyond the scope of the current study. Future work incorporating ICS with appropriate lineage gating would help further delineate the cytokine-secreting contributions of individual regulatory populations within the DSIMC.

Thymidine incorporation assays were used as functional indicators of DSIMC suppressive capacity, as previously established in earlier studies.^5,26,52^ This method clearly demonstrated a dose-dependent suppressive effect of the generated cells on mixed lymphocyte culture (MLC) and has been widely used as a gold standard for *in vitro* proliferation testing since its introduction in the 1960s.^53^ However, it cannot identify the responding cell populations, providing only a bulk measure of proliferative activity across all cells present in the culture. Alternative flow cytometry-based proliferation assays were therefore explored. Amongst these, CFSE and violet cell trace staining were tested, but the presence of three distinct cell populations in co-culture made it difficult to distinguish proliferating responder cells from non-stained generated cells in cell trace staining. Ki67 staining was also examined after seven days of culture, as it marks cells across all phases of the cell cycle (G1, S, G2, and mitosis).^54^ However, Ki67 positivity in responder cells ranged between 3 and 25% following donor antigen stimulation, and co-culture with generated cells could paradoxically increase this proportion, likely because the generated cells themselves express Ki67, precluding reliable distinction of suppressive effects. Palutke et al. reported that Ki67 staining correlated with thymidine incorporation data and could serve as a replicable method,^55^ while Lastovicka et al. found good correlation between CFSE and Ki67 but weaker correlation with thymidine incorporation results.^56^ Given the dose-dependent clarity of the thymidine incorporation assay in our system and the interpretive challenges of the alternatives, we retained this method as the primary functional readout, while using Ki67 to compare the effects of different DSIMC subpopulations in a relative sense.

B cell-depletion of DSIMC did not alter the immunomodulatory function compared to non-sorted DSIMC in thymidine incorporation assays. Interestingly, our results from Ki67 staining suggested that B cells may play a role in suppressing T cell proliferation more generally, beyond antigen-specific responses. Although previous reports suggest that macrophages and dendritic cells have subpopulations that have regulatory functions, these innate cell populations and Treg populations would have to interact with each other to create a tolerogenic environment.^19,42,57–59^ However, our method of culturing DSIMC generates a product with a minimal amount of these kinds of APCs, and depletion or enrichment of the innate cell populations was therefore not feasible in this study. In this setting, it is possible that B cells act as the predominant APCs, and depletion of this population may therefore decrease antigen-specific responses. The influence of DSIMC on recipient macrophages has been shown in an experimental mouse model.^57^ In the article. it is demonstrated that the co-injection of DSIMC may suppress severe inflammation caused by pancreatic islet cell transplantation to improve engraftment, suggesting a different treatment indication for DSIMC.

Although Tregs are currently viewed as the primary cell population driving immunomodulation, self-tolerance and donor-specific immune acceptance in allogeneic transplantation settings, other regulatory subsets have been increasingly recognized as contributors to these processes. Tr1 cells are of particular interest in the context of the current study, as the IL-10 and IFN-γ co-production observed in CD4^+^ populations of the DSIMC following donor antigen stimulation may be consistent with a Tr1-like contribution,^60^ though formal characterization of this subset was beyond the scope of the current study. Regulatory B cells, CD8^+^ regulatory T cells, and regulatory dendritic cells have also been proposed to contribute to tolerance induction, though their relative contributions remain incompletely understood. Currently, few tolerance induction protocols have successfully achieved complete immunosuppression (IS) weaning, while many have demonstrated promise in reducing the long-term IS burden.^6,11,61–63^ In Todo et al.’s study,^9^ a majority of patients achieved complete IS withdrawal. Importantly, although the cell product was described as containing Tregs, no formal Treg enrichment or purification was performed, and the product contained multiple cell lineages exposed to donor antigens. The fact that several protocols employing purified regulatory cell populations have not replicated this degree of operational tolerance suggests that tolerance induction, like many complex immunological processes, may be orchestrated through the coordinated activity of multiple cell populations. The results from the current study support this hypothesis.

The ultimate aim of identifying the key immunomodulatory components of DSIMC would be to enable their enrichment or purification, thereby optimizing the donor-specific immunomodulatory effect while minimizing unwanted cell populations, an important consideration for GMP-compliant clinical production. Given that CD4^+^CD25^+^CD127^-/low^FoxP3^+^ Tregs are currently regarded as central mediators of transplant tolerance, we applied available enrichment strategies targeting this population. MACS-based enrichment using CD8 depletion followed by CD25 selection has been employed in clinical Treg therapy protocols, including in pancreatic islet transplantation^60^ and kidney transplantation.^64^ FACS-based purification of CD4^+^CD25^+^CD127^-/low^ cells has also been explored as an enrichment strategy, demonstrating efficacy in a humanized mouse model.^65^ However, a well-recognized limitation of CD25-based enrichment is the co-selection of recently activated effector T cells, which also upregulate CD25, making FoxP3 quantification necessary to confirm Treg purity in the resulting product. This limitation remains difficult to fully address, especially within the currently accepted GMP-compliant cell manipulation methods and represents an ongoing challenge for the clinical translation of Treg-based cell therapy protocols.^66^

## Conclusion

The regulatory cell therapy protocols hold promise for minimizing, and in optimal cases enabling complete withdrawal of, lifelong immunosuppression after solid organ transplantation. The results of this study demonstrate that the donor-specific immunomodulatory efficacy of DSIMC resides not within a single purifiable regulatory subset but emerges from the collective activity of multiple interacting cell populations. Enrichment or depletion of individual cell lines did not enhance, and in several cases diminished donor-specific immunomodulatory function. These findings highlight the importance of preserving the cellular complexity of these DSIMC during product development. Further mechanistic studies, including single-cell characterization of regulatory subsets and cytokine profiling at the cellular level, are needed to fully elucidate the basis of donor-specific immunomodulation and to guide rational optimization of cell therapy protocols for clinical tolerance induction.

## Supplemental material

Supplemental material - Donor-specific immunomodulation is mediated by mechanisms beyond CD4^+^ regulatory T cells Supplemental material for Donor-specific immunomodulation is mediated by mechanisms beyond CD4^+^ regulatory T cells by Makiko Kumagai-Braesch, Nils Ågren, Masaaki Watanabe, Bo-Göran Ericzon, Michael Uhlin, Ming Yao in Cell Transplantation
