# Supplementary material for: Donor-specific immunomodulation is mediated by mechanisms beyond CD4+ regulatory T cells
Source: Cell Transplant. 2026 Aug 3;35:09636897261473440. doi: 10.1177/09636897261473440 (PMC13434093; doi:10.1177/09636897261473440)
Supplement: Supplementary file 1 [file sj-pdf-1-cll-10.1177_09636897261473440.pdf]

Supplemental Figure 1

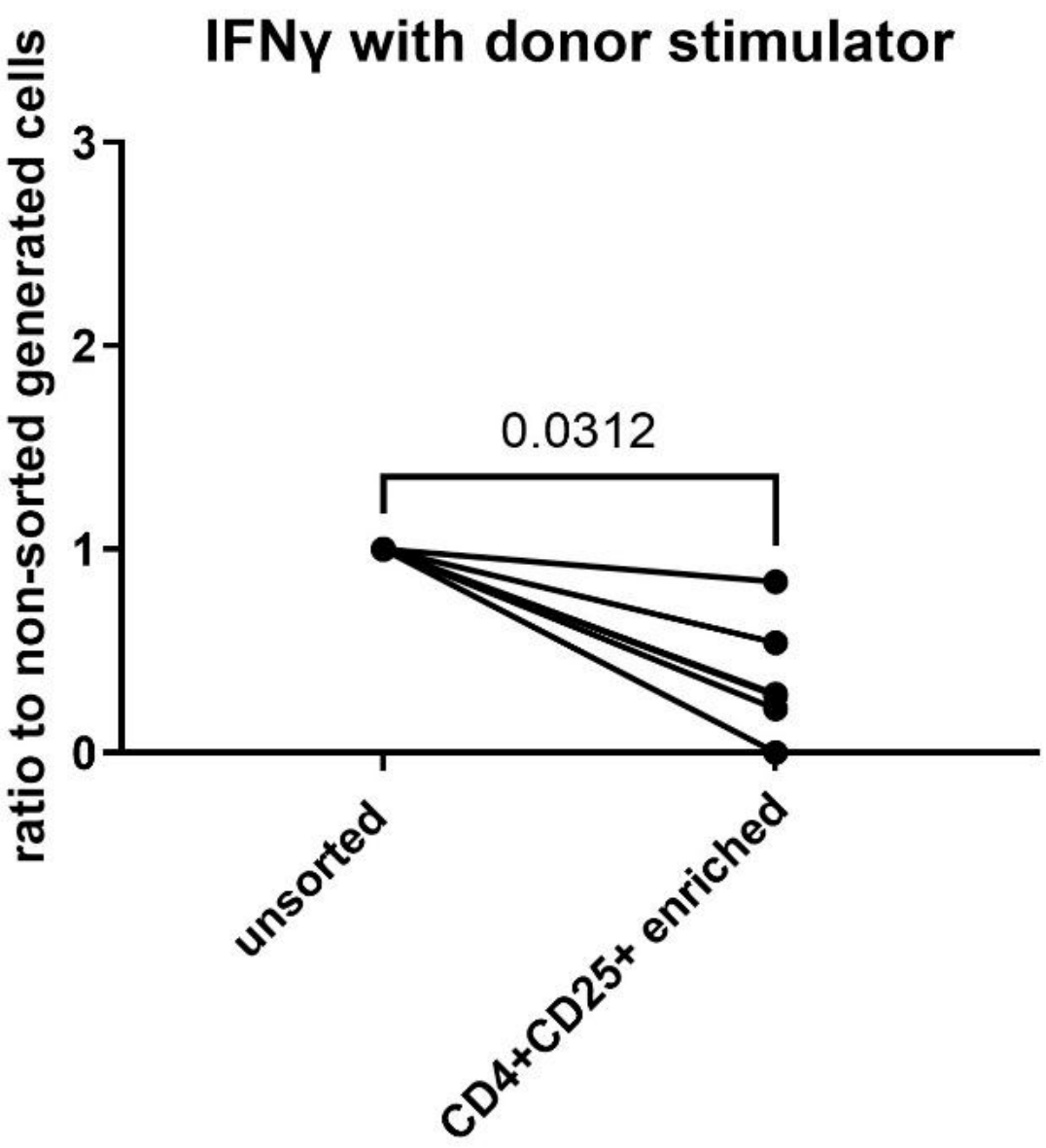

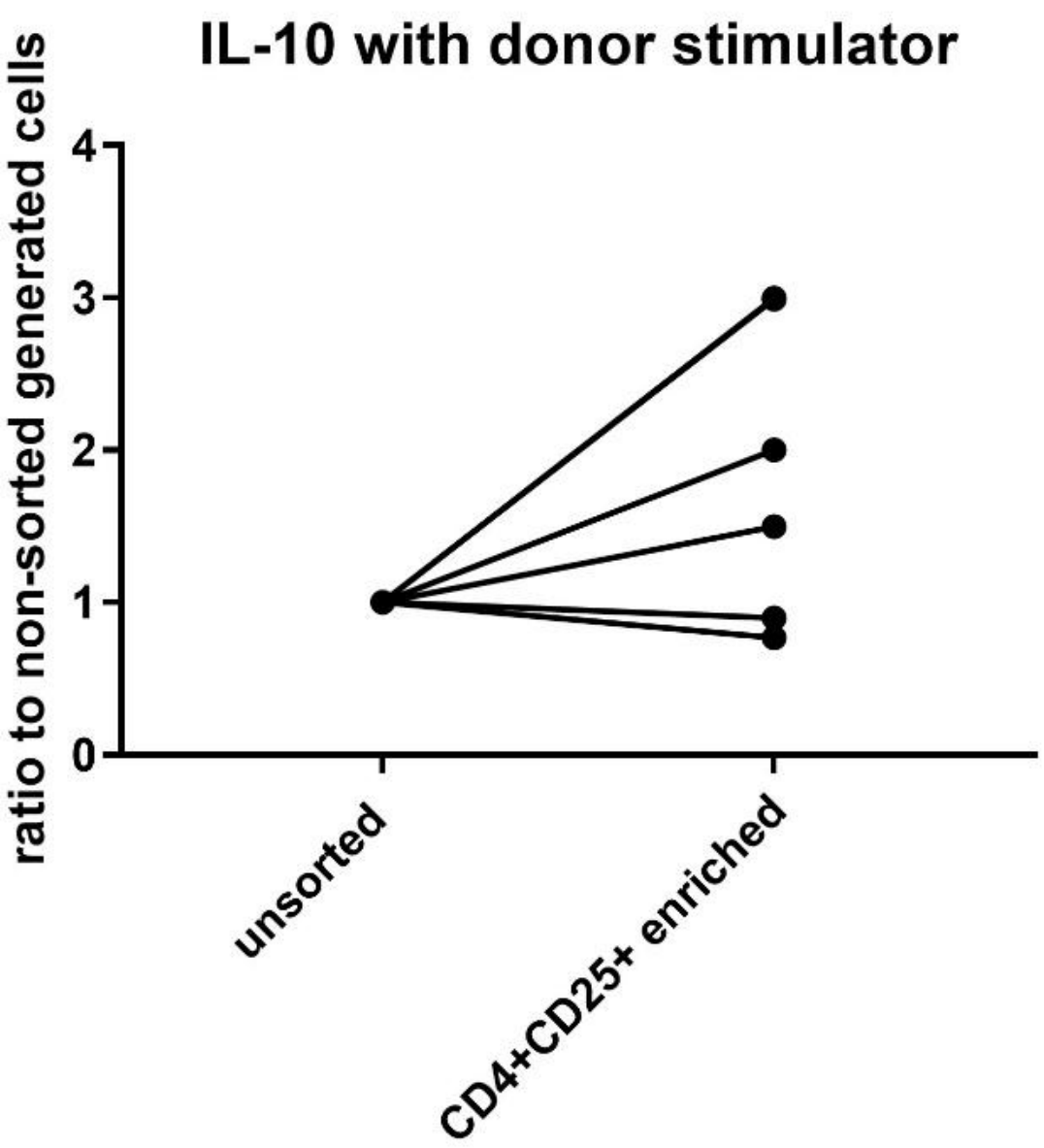

IFN-γ and IL-10 producing cell numbers after donor specific antigen-stimulation: Detected by ELISpot assay.

The non-separated whole generated cells and $CD4^+CD25^+$ enriched generated cells were stimulated with irradiated donor with a stimulator cell-generated cell ratio of 1/0.25 for 24 hours. Spot-forming unit was compared. The Treg-enriched population produced significantly lower IFN-γ and tended to increase IL-10 production.

n=5 Wilcoxon tests

Supplemental Figure 2

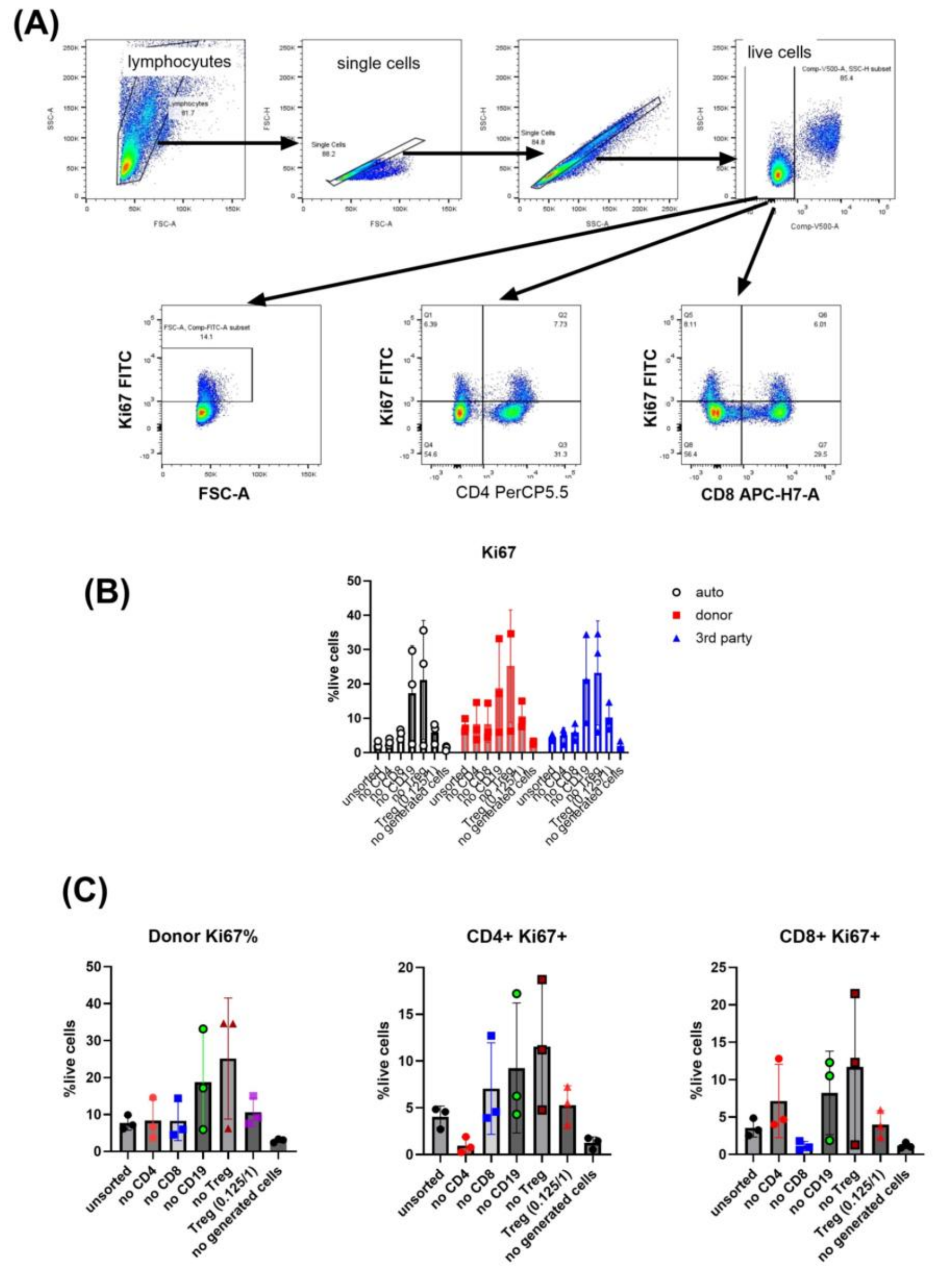

Supplemental Figure 2. Ki67 staining as indicates of proliferating cells.
DSIMC were sorted on day 14, by depleting CD4 (no CD4) CD8 (no CD8) CD19+ B cells (no CD19), and CD25+CD127- cells (no Treg). CD25+CD127- cells (Treg) were also collected. Each fraction was added to mixed lymphocyte culture (MLC) with responder/generated cell ratio of 1/0.5. Due to a few number pof cells, Treg was added 1/0.125. On day 7, cells were harvested and Ki67 expression was examined. (A) showed gating strategy for Ki67 + cells. Live cells were gated and Ki67+ cells in total live cells. (B)% of Ki67+ cells in MLC, with autologus, specific donor, or 3rd party stimulators. Ki67 expression was higher than MLR without DSIMC. Depletion of CD4 or CD8+ T cells did not significantly changed % expresion of Ki67 compared to non sorted group. DSIMC depleted of CD19 and Treg exhibited higher % of Ki67, though due to high variation, there was no significant differences. n=3
